# Surgical strategies for intracranial meningioma in the molecular era

**DOI:** 10.1007/s11060-023-04272-z

**Published:** 2023-04-03

**Authors:** Alper Dincer, Saul F. Morales-Valero, Stephanie M. Robert, Joanna K. Tabor, Joseph O’Brien, Kanat Yalcin, Robert K. Fulbright, Zeynep Erson-Omay, Ian F. Dunn, Jennifer Moliterno

**Affiliations:** 1 Department of Neurosurgery, Tufts Medical Center, Boston, MA USA; 2 Department of Neurosurgery, Yale School of Medicine, 15 York Street, LLCI 810, New Haven, CT 06510 USA; 3 The Chenevert Family Brain Tumor Center, Smilow Cancer Hospital, New Haven, CT USA; 4 Department of Radiology and Biomedical Imaging, Yale School of Medicine, New Haven, CT USA; 5 Department of Neurosurgery, Oklahoma University Medical Center, Oklahoma City, OK USA

**Keywords:** Meningioma, Surgery, Genetics, Tumor

## Abstract

**Introduction:**

Surgical resection has long been the treatment of choice for meningiomas and is considered curative in many cases. Indeed, the extent of resection (EOR) remains a significant factor in determining disease recurrence and outcome optimization for patients undergoing surgery. Although the Simpson Grading Scale continues to be widely accepted as the measure of EOR and is used to predict symptomatic recurrence, its utility is under increasing scrutiny. The influence of surgery in the definitive management of meningioma is being re-appraised considering the rapid evolution of our understanding of the biology of meningioma.

**Discussion:**

Although historically considered “benign” lesions, meningioma natural history can vary greatly, behaving with unexpectedly high recurrence rates and growth which do not always behave in accordance with their WHO grade. Histologically confirmed WHO grade 1 tumors may demonstrate unexpected recurrence, malignant transformation, and aggressive behavior, underscoring the molecular complexity and heterogeneity.

**Conclusion:**

As our understanding of the clinical predictive power of genomic and epigenomic factors matures, we here discuss the importance of surgical decision-making paradigms in the context of our rapidly evolving understanding of these molecular features.

## Introduction

Meningiomas are the most common primary brain tumors, accounting for nearly 40% of intracranial tumors [1]. When intervention is necessary, due to size and/or symptomatology, the mainstay of treatment is maximal safe resection. Surgical intervention alleviates mass effect, relieves associated neurological symptoms, decreases risk of recurrence, and provides for diagnosis and molecular characterization. Gross total resection (GTR) is often considered curative in many cases [2]. Radiotherapy can be used as a stand-alone approach and more commonly as an adjunct for more aggressive lesions. There are currently no effective standard pharmacological therapies.

Meningiomas are divided into three World Health Organization (WHO) grades (Grades 1–3) based on histology. Grade 1 tumors are often considered benign tumors and are the most common, accounting for 80% of all meningiomas and demonstrate relatively low risk of recurrence. WHO grade 1 meningiomas have historically and generally been considered a “benign” tumor type, one that was readily cured through surgical resection. Grade 2 and 3 meningiomas are higher-grade tumors that can exhibit increased growth and recurrence rates > 70% following GTR, as well as increased mortality despite multimodal therapy [3, 4].

**Fig. 1:**
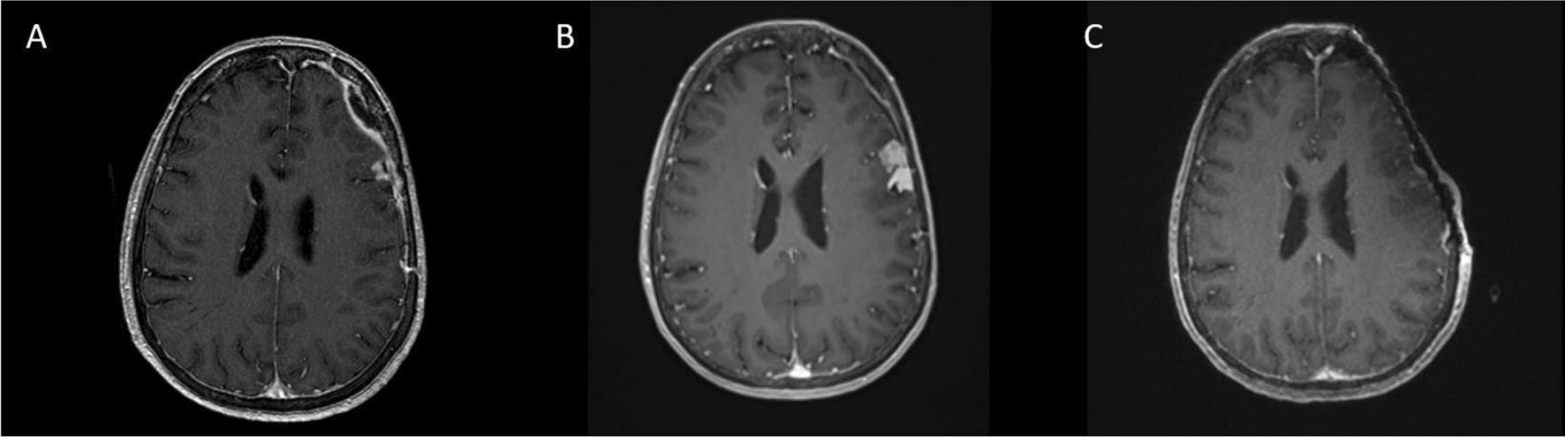
A 70-year-old female underwent resection of a left convexity meningioma at an outside institution. A subtotal resection was achieved. The residual tumor was left adherent to the brain (**A**) and the initial diagnosis was meningioma WHO grade 1. She was then referred to us given the progression of residual tumor (**B**); re-evaluation of initial pathology was concerning for anaplastic meningioma WHO grade 3. She underwent an aggressive resection of the entirety of the tumor, including the involved dura and bone, as well as mesh cranioplasty (**C**)

As our understanding of meningioma natural history has progressed, these tumors are increasingly recognized as a significantly more complex and heterogenous group. Indeed, meningiomas do not always behave in accordance with their WHO grade, such that histologically-confirmed grade 1 tumors can demonstrate unexpected recurrence, malignant transformation, and aggressive behavior, more likely expected with that atypical or anaplastic meningioma [5, 6]. Higher-grade WHO tumors may, conversely, behave and appear in a more benign manner (Fig. 1). Additionally, meningioma recurrence rates demonstrate wide variations across both EOR and tumor grade. Similar recurrence rates have been reported between subtotal resection (STR), GTR, as well as different tumor grades [7, 8].

**Fig. 2:**
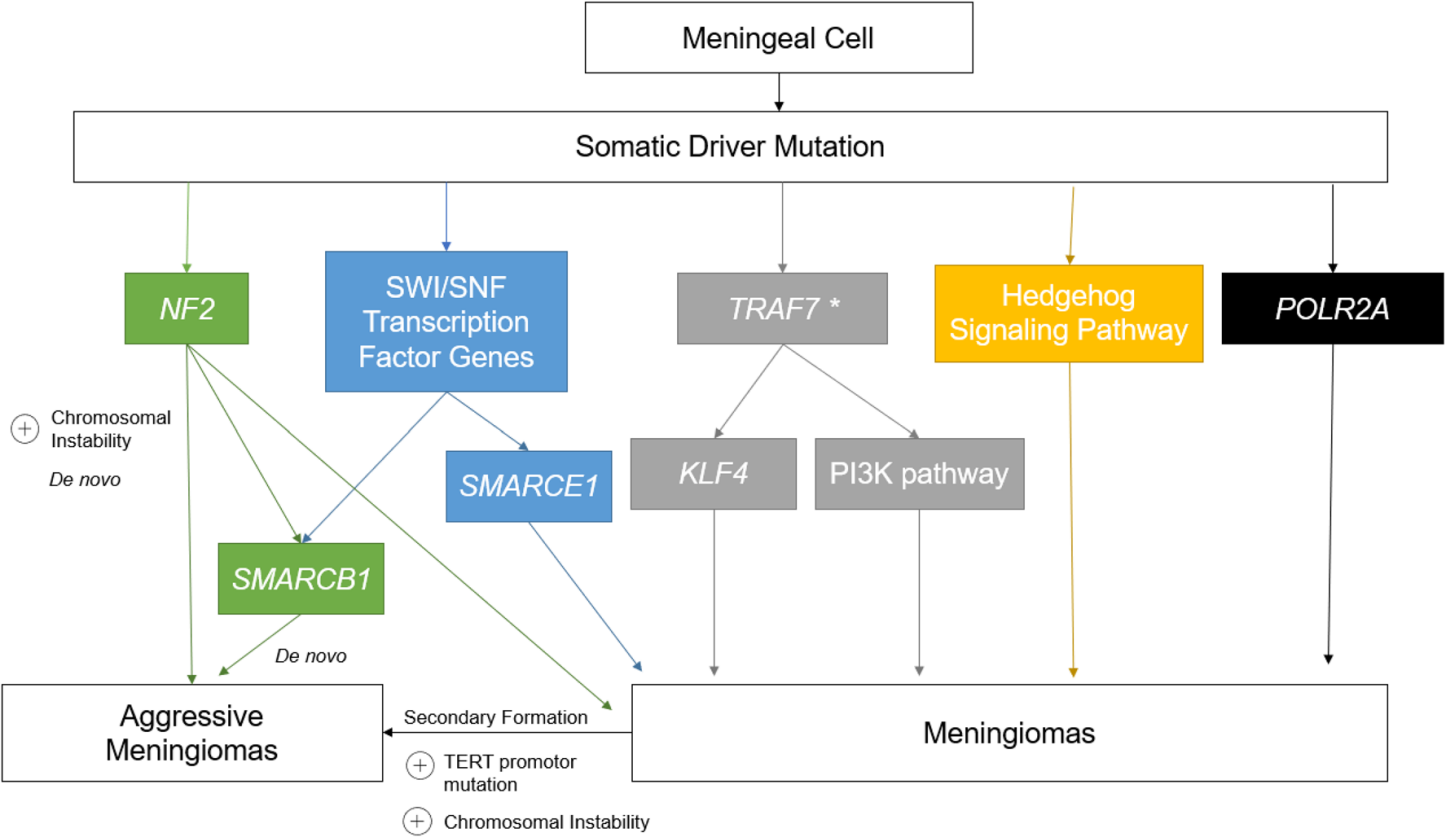
Meningioma driver mutations and pathways to aggressive meningioma formation. Genomic subgroups are based on somatic alterations in the following genes: (1) *NF2* (and/or Chr22q loss); (2) *SMARCB1* with or without *NF2*; (3) *KLF4* with or without mutant *TRAF7*; (4) PI3K signaling, with or without mutant *TRAF7*; (5) Hedgehog signaling pathway mutation; (6) *POLR2A* mutation; or (7) *TRAF7* without *KLF4* or PI3K signaling mutations. *TRAF7 mutations appear to be necessary but not sufficient to cause meningioma formation. Rarely, *TRAF7* mutations can be found independently, but this is most likely due to co-mutations yet to be discovered. Adapted from Gupte et al. [11]

These discordant observations in natural history and aggression of meningiomas are likely explained by their differences in molecular biology. Indeed, the last decade has afforded a robust understanding of the genomic and epigenetic landscape of meningiomas and correlations with clinical variables and outcomes [9, 10]. Recently identified somatic driver mutations have defined at least seven clinically relevant molecular subgroups (*NF2, POLR2A, SMARCB1, TRAF7, KLF4*, molecules involved in the Hedgehog and PI3K pathway) that demonstrate differences in appearance, intracranial location, natural history, and recurrence after resection. These driver mutations, as well as chromosomal instability, copy number variations (CNVs), and DNA methylation patterns have created a paradigm shift in our understanding of the behavior of meningiomas (Fig. 2).

Accordingly, the recently updated WHO grading of meningiomas now considers and incorporates molecular data along with histological diagnosis [12]. As our understanding of meningiomas and classification of them has evolved, here we review how this new knowledge may influence overall treatment decisions and the role of surgery.

## Indications for meningioma surgery

Current initial treatment strategies for meningiomas include observation, surgical resection, and/or radiotherapy. Among these, radiation is widely accepted as a stand-alone treatment for a presumed meningioma or as an adjunct after surgery, but not within the scope of this review. Certainly, surgery is not indicated for all meningiomas. A range of factors impact the decision for surgical management and timing of intervention, including presenting symptoms, patient age, medical comorbidities, and tumor characteristics, such as size, the presence of edema and/or mass effect in the surrounding brain.

Intracranial meningiomas can often be incidentally found, more recently with increasing incidence. This is most likely due to an aging population and more frequent utilization of more sensitive brain imaging [13–16]. Studies report the presence of one or more meningiomas on magnetic resonance imaging (MRI) scans is nearly 1% of the general population [15, 17]. While there is no class I or II evidence to support a standard protocol, incidental, asymptomatic, small to moderate sized meningiomas without concerning radiographic features (discussed below) are generally monitored with serial imaging. The reported growth rates of untreated, incidental meningiomas, however, vary greatly. Growth has been reported in 11% to 74%, of patients, of which 0% to 56% become symptomatic [18–20]. The majority of tumors grow less than 1 cm^3^/year, but growth can range anywhere from 0.03 to 2.62 cm^3^/year [21]. These wide ranges underscore the relative biologic heterogeneity of meningiomas, emphasizing the importance of management on a patient-by-patient basis.

**Fig. 3:**
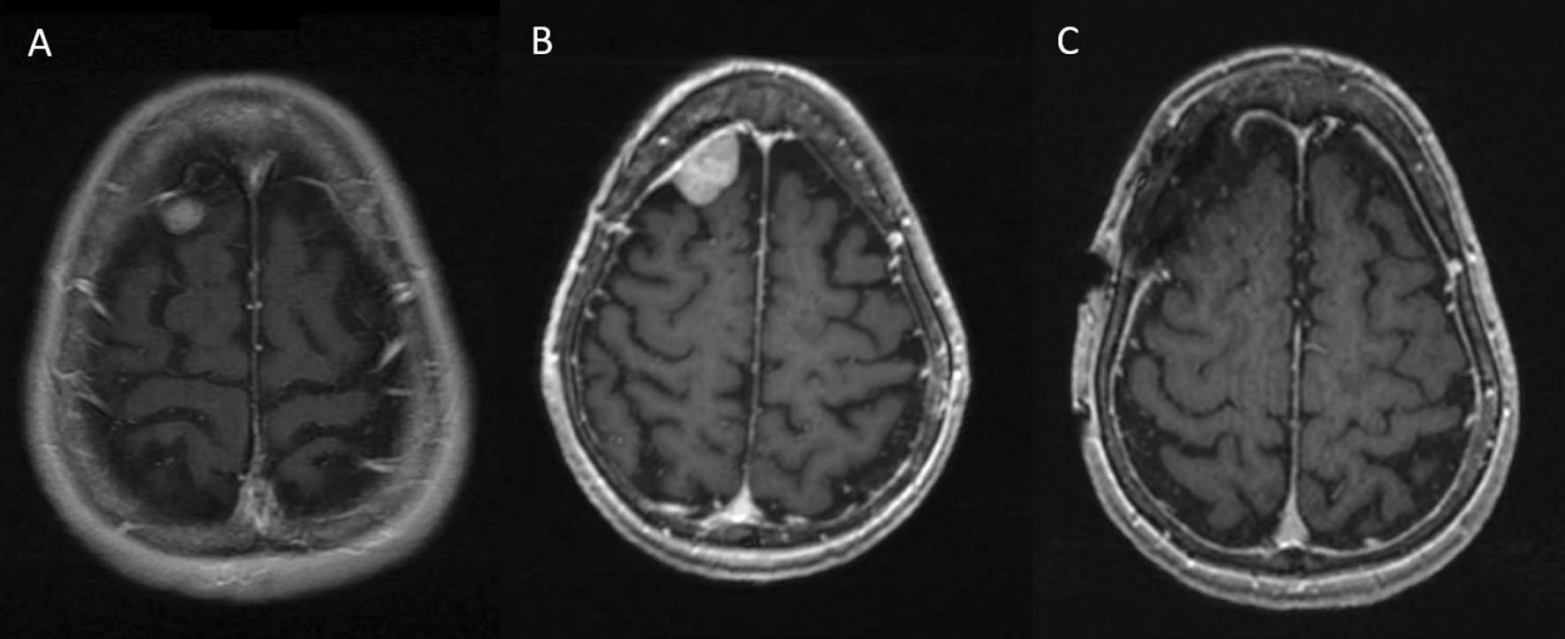
78-year-old female who initially presented with symptoms of dizziness, and T1 MRI with contrast demonstrated an extra-axial lesion measuring 1.4 × 0.8 × 0.8 cm (**A**). The lesion was presumed to be a meningioma and no resection was recommended at the time. Two years later, serial imaging demonstrated an increase in size of the mass (**B**). The patient underwent gross total resection (**C**), and pathology confirmed a WHO grade 2, atypical meningioma. Genomic report demonstrated an aggressive profile, with an NF2 somatic driver mutation and multiple copy number variants including chr1p deletion, chr6 deletion, chr18 deletion, chr19p deletion, and chr22 (NF2) deletion. She completed a course of adjuvant radiation therapy

Enlargement in meningioma size, and especially at an accelerated rate, typically warrants consideration for surgical intervention (Fig. 3). Meningiomas arising from certain locations, such as those abutting the optic nerve near the optic canal, can often require earlier intervention at smaller sizes given their proximity to cranial nerves and their symptomatology. Moreover, specific radiographic features, including heterogenous signal with evidence of necrosis, irregular contrast enhancement, bony involvement, and extensive peritumoral edema, are thought to indicate a more aggressive tumor [22, 23].

We must keep in mind that surgical decision-making has historically been based on the natural history of “benign” meningiomas. However, benign-appearing and even histologically benign meningiomas may have molecular characteristics predisposing to aggressive behaviors. As we gain further insights into the impact of meningioma biology on clinical behavior, molecular characteristics need to be incorporated into the decision-making paradigm for meningiomas. Determination of meningioma molecular architecture prospectively prior to surgery is becoming increasingly feasible (discussed below) and can aid in our prediction of tumor aggression and natural history of meningiomas. In this way, molecular characteristics of meningiomas can be used to aid in surgical decision making.

## Extent of surgical resection and outcome

Meningiomas can be challenging surgical lesions for a variety of reasons. Preliminary pathology at the time of surgery does not reliably define the aggressiveness of the tumor, and therefore, extent of resection (EOR) goals must be decided based on clinical, imaging, and intraoperative features. Brain invasion or bony involvement, for instance, may indicate a more aggressive underlying tumor biology, and support more aggressive resection when safe.

Once a surgery decision is made, the goal of meningioma surgery remains maximally safe resection. EOR has repeatedly been shown to be directly associated with improved progression-free survival (PFS) and is one of the most significant factors to influence disease recurrence and patient outcomes [24]. Cushing concluded in 1938 that while the GTR of a meningioma is often curative, seemingly benign, local areas of disease infiltration were likely the source of recurrence. Accordingly, maximal safe EOR for meningiomas often requires removal of tissue beyond the tumor boundary, including the surrounding dura and overlying bone.

Based on these observations, Donald Simpson created a grading scale in 1957, which relates the degree of EOR with symptomatic recurrence (Table 1) [24]. The Simpson grading scale includes five grades (grades I–V) that describe varying degrees of surgical resection and prediction of recurrence for WHO grade 1 meningiomas. Simpson grades I–III are considered GTRs, while grades IV and V are subtotal. Simpson grade I denotes the macroscopic complete resection of the meningioma along with associated bone and dura, correlating with a 9% risk of recurrence 10 years following surgery. Coagulation of the dura, rather than its removal, is classified as a Simpson grade II resection and is associated with a 19% risk of symptomatic recurrence. GTR without resection or coagulation of surrounding dura is a Simpson grade III and is associated with a 29% risk of symptomatic recurrence. Simpson grades IV and V are STRs and correlate with increased symptomatic recurrence at 10 years following surgery ranging from 44 to 100% [24].

**Table 1:** Simpson grading scale demonstrating the incidence of symptomatic recurrence at 10 years after surgical treatment

Grade	Definition	Symptomatic recurrence at 10 years (%)
I	Macroscopic GTR with excision of dural attachment and underlying bone	9
II	Macroscopic GTR with coagulation of dural attachment	19
III	Macroscopic GTR without resection or coagulation of dural attachment	29
IV	Subtotal resection	44
V	Simple decompression with or without biopsy	100

While generally accepted as the “gold standard” for predicting recurrence of WHO grade 1 meningiomas based on EOR, many limitations have been described to the Simpson grading scale [7, 8, 25, 26]. Most significant is the subjectivity of the scale, which relies on the surgeon’s intraoperative assessment of EOR. Simpson grade I resections are arguably the most straightforward to grade and yet they are associated with a wide range of associated recurrence rates from 10 to 55% in the literature [27–29]. Furthermore, studies attempting to validate the scale have only demonstrated clear correlations between GTR (Simpson grade I–III) and STR (Simpson grade IV–V) with recurrence rates, rather than a more granular association of Simpson grade with increased risk of recurrence [25, 26, 30].

Interestingly, skull base meningiomas demonstrate significant deviation from this grading scale, as it has been reported that their recurrence rates do not correlate with EOR [31, 32]. Although WHO grade 1 lesions are more commonly found in skull base locations [33], the risk of STR is higher given challenges of resection in this area due to neurovascular structure involvement. Despite this, the risk of recurrence of skull base tumors was found to be similar for Simpson grade I, II, and III resections (11–14%). In a large retrospective study including 325 skull base lesions, there was no demonstrated significant difference in recurrence for olfactory groove, sphenoid wing, petroclival, and cerebellopontine angle meningiomas based on either Simpson grade or EOR [31].

This finding corroborates studies that demonstrate skull base tumors harbor more benign mutations, including *TRAF7*, *KLF4*, *AKT1*, and *SMO* genes [33], compared to cerebral and cerebellar tumors whose recurrence and/or progression more directly correlates with EOR. This is important, as it argues that aggressive surgical resection does not improve patient outcomes in these more dangerous, skull base locations. Conversely, non-skull base lesions (i.e. convexity, parafalcine), frequently demonstrate more aggressive somatic driver mutations, including *NF2* mutant tumors, but given their location, these lesions are typically more amenable to GTR [31]. These findings further demonstrate how applying an understanding of meningioma genomics can improve surgical decision making and maximize safe EOR in meningioma surgery.

The Simpson grading scale also does not apply to higher-grade meningiomas, which generally demonstrate more aggressive clinical behavior and higher regrowth despite maximal EOR [30]. These WHO grade 2 and 3 lesions demonstrate variable recurrence rates, ranging from 9 to 50% after GTR and 36 to 83% after STR [34]. This variability suggests that there are likely underlying biological factors that influence outcome irrespective of EOR [35]. However, STR does correlate with decreased overall survival (OS) compared to GTR, with 5-year OS rates 78.2% vs. 91.3% for WHO grade 2 meningiomas and 41.1% vs. 64.5% for WHO grade 3 meningiomas [36]. Despite the variability in reported recurrence rates, the literature demonstrates a clear survival benefit in achieving maximally safe resection in meningioma surgery for higher-grade tumors.

## Prediction of molecular make-up prior to initial meningioma surgery as a guide

Recent advances in molecular genomics have defined mutually exclusive meningioma subtypes, with established prognostic implications and clinical relevance. Similar to meningioma histological grading, the molecular profile is also not available prior to or during the initial surgery. However, the underlying driver mutation may be predicted based on radiographic features, such as tumor location [9] and bony involvement [33, 37, 38], as well as clinical presentation [11].

**Fig. 4:**
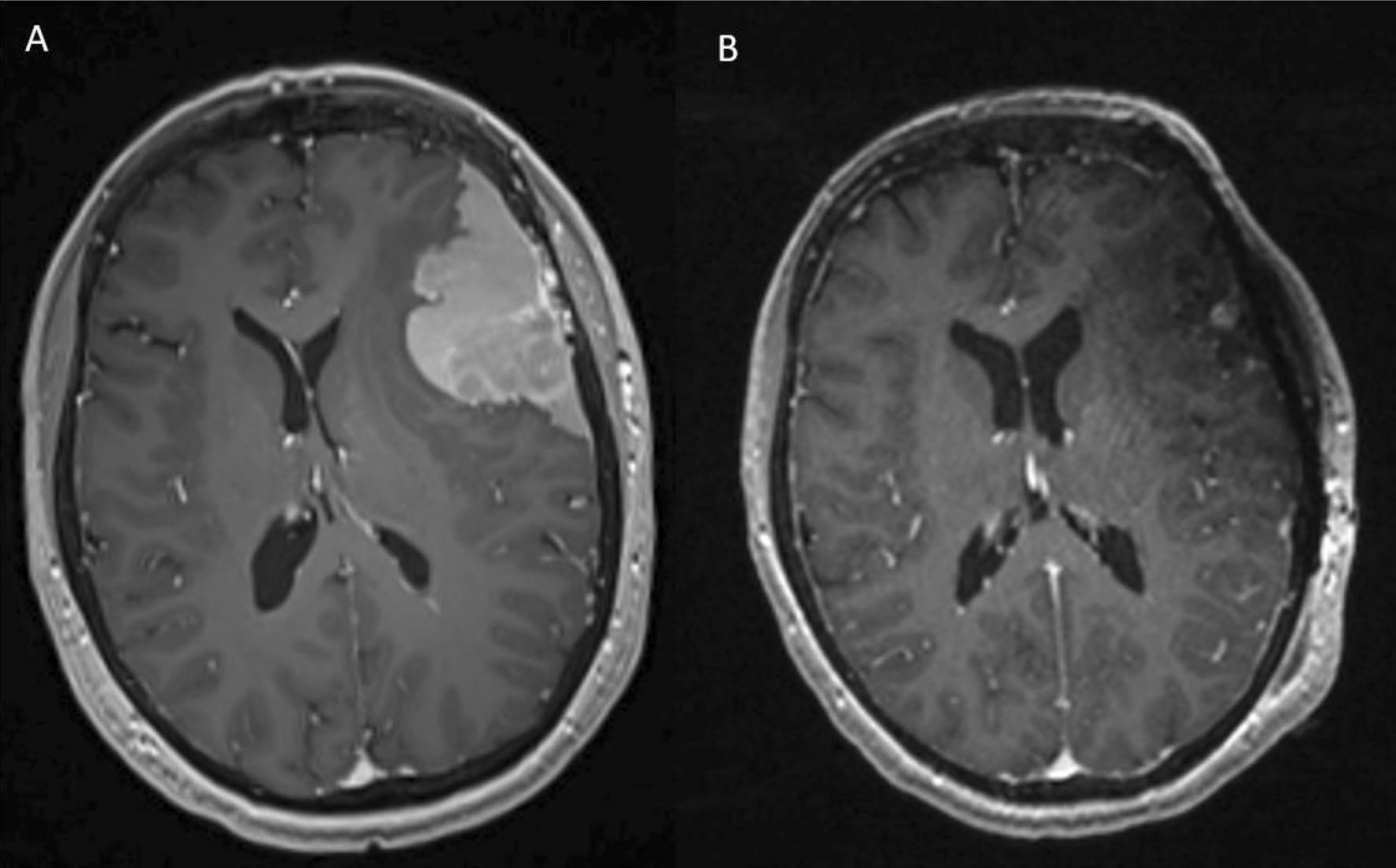
A 60-year-old right-handed female presented with an episode of word-finding difficulty. Axial MRI with contrast demonstrates an enhancing extra-axial lesion in the left frontotemporal convexity causing mass effect and surrounding edema (**A**). There is concern for bone invasion, which was confirmed intraoperatively. The patient underwent a Simpson grade I resection and mesh cranioplasty (**B**). Pathology confirmed a grade 1 meningioma with NF2 mutation

**Fig. 5:**
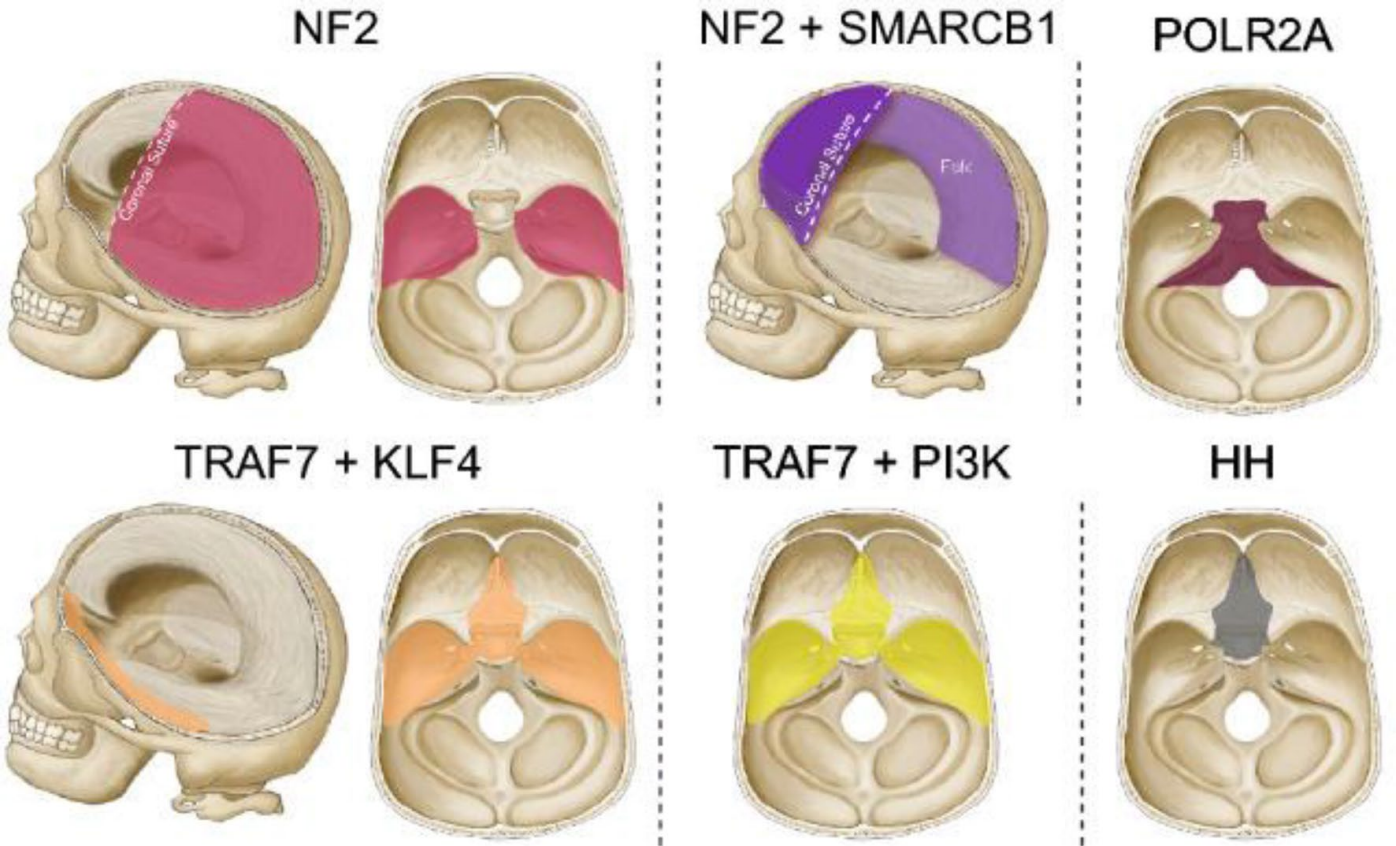
Schematic representation of the spatial association of the main genomic subgroups of meningiomas with their anatomical location

*NF2* mutant meningiomas, for instance, typically originate from lateral segments, along the cerebral convexities or skull base (Figs. 4, 5). *NF2*-mutated tumors also follow an anterior–posterior gradient, more commonly arising posterior to the coronal suture (Fig. 5) [9]. Rarely, *NF2* mutant meningiomas are found more midline, in the parafalcine region, and harbor somatic co-mutations in the SWI/SNF transcription factor gene, *SMARCB1* [9, 33].

**Fig. 6:**
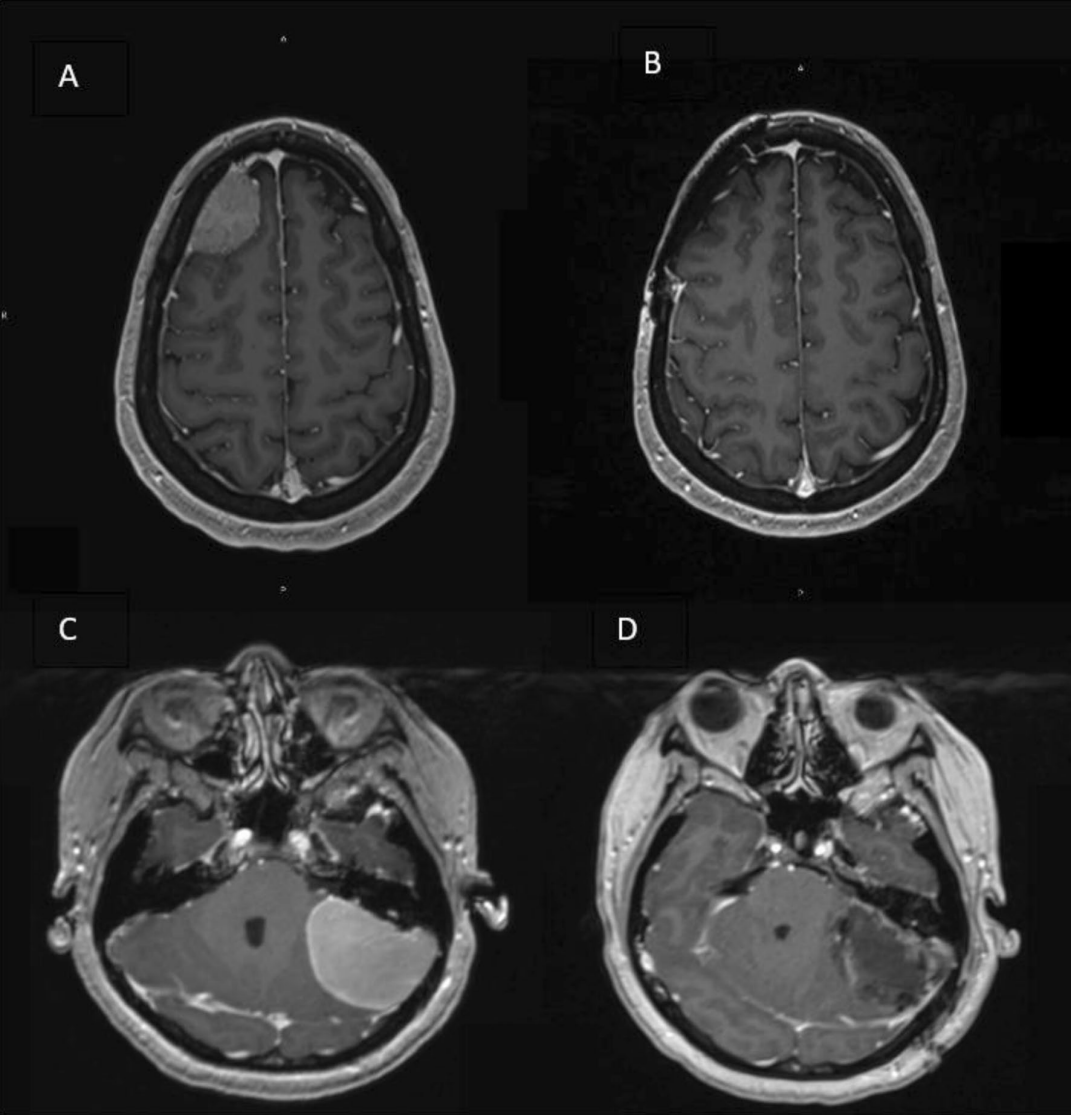
Axial views of an MRI with contrast demonstrating an *NF2* subtype meningioma of the convexity before (**A**) and after gross total resection (**B**) and a large *NF2* subtype posterior fossa meningioma before (**C**) and after (**D**) gross total resection. Despite its more benign appearance, the convexity meningioma (**A**, **B**) is a WHO grade 2 atypical tumor with NF2 mutation as well as a somatic *SUFU* mutation, genomic instability, and 65% of genome loss of heterogeneity. Conversely, the posterior fossa meningioma (**C**, **D**) appears more aggressive but is a WHO grade 1 tumor without genomic instability

Although from a surgical perspective, *NF2* mutant meningiomas may be regarded as “simpler” as they tend to originate in more surgically accessible locations, such as the convexity, they are the biologically more aggressive tumors and more likely to become atypical and malignant [11]. Indeed, they are more commonly associated with higher proliferative (Ki-67) index, WHO grade, brain invasion, and peritumoral edema [39]. Patients who harbor *NF2* mutant meningiomas are typically male, present with preoperative seizures and have larger tumors (Fig. 6) [11]. Postoperatively, *NF2* mutant tumors are associated with higher rates of recurrence and undergo post-operative radiotherapy more frequently than non-NF2 mutant meningiomas [9, 10]. Therefore, a convexity meningioma in an asymptomatic patient should perhaps be followed more closely with a lower threshold for surgical intervention. Moreover, surgery should be aimed at safely removing all involved tissue with a wide dural resection and removal of bone with cranioplasty, when possible.

Meningiomas in the midline anterior skull base, along the olfactory groove or planum sphenoidale, typically harbor an underlying somatic mutation in one of the molecules involved in Hedgehog signaling, including *SMO* or *SUFU* [9, 33, 40]. These tumors are among the largest at presentation, often associated with varying degrees of hyperostosis of the skull base. The biological significance of the presence of hyperostosis is unclear and its complete removal is often not pursued as it can risk CSF leak. However, while meningiomas with mutations in *SMO* and *SUFU* are more commonly associated with lower-grade histology, they have been found to have higher rates of recurrence [10, 40], possibly related to bony invasion as evidenced by hyperostosis. Cases in which a decision is made as not to remove the bony involvement should therefore be watched with close follow-up to assess for recurrence.

Other non-*NF2* mutant tumors, including those with *TRAF7* mutations co-occurring with a recurrent mutation in *KLF4* or a mutation involving one of the PI3K pathway molecules typically localize to the middle cranial fossa, and most often along the sphenoid wing [9, 33]. Sphenoid wing meningiomas harboring *NF2* mutations tend to demonstrate bony invasion with frank tumor, while *TRAF7*-mutant tumors are associated with hyperostosis [41]. Those tumors with mutations involving the PI3K signaling pathway, such as *PI3KCA* or more commonly *AKT1*, have been found to show early recurrence [10]. Removal of tumors in this location, particularly along the medial sphenoid wing, which often encase critical neurovascular structures, such as the internal carotid artery and the optic nerve, often limit extent of safe resection, potentially contributing to these early recurrences.

**Fig. 7:**
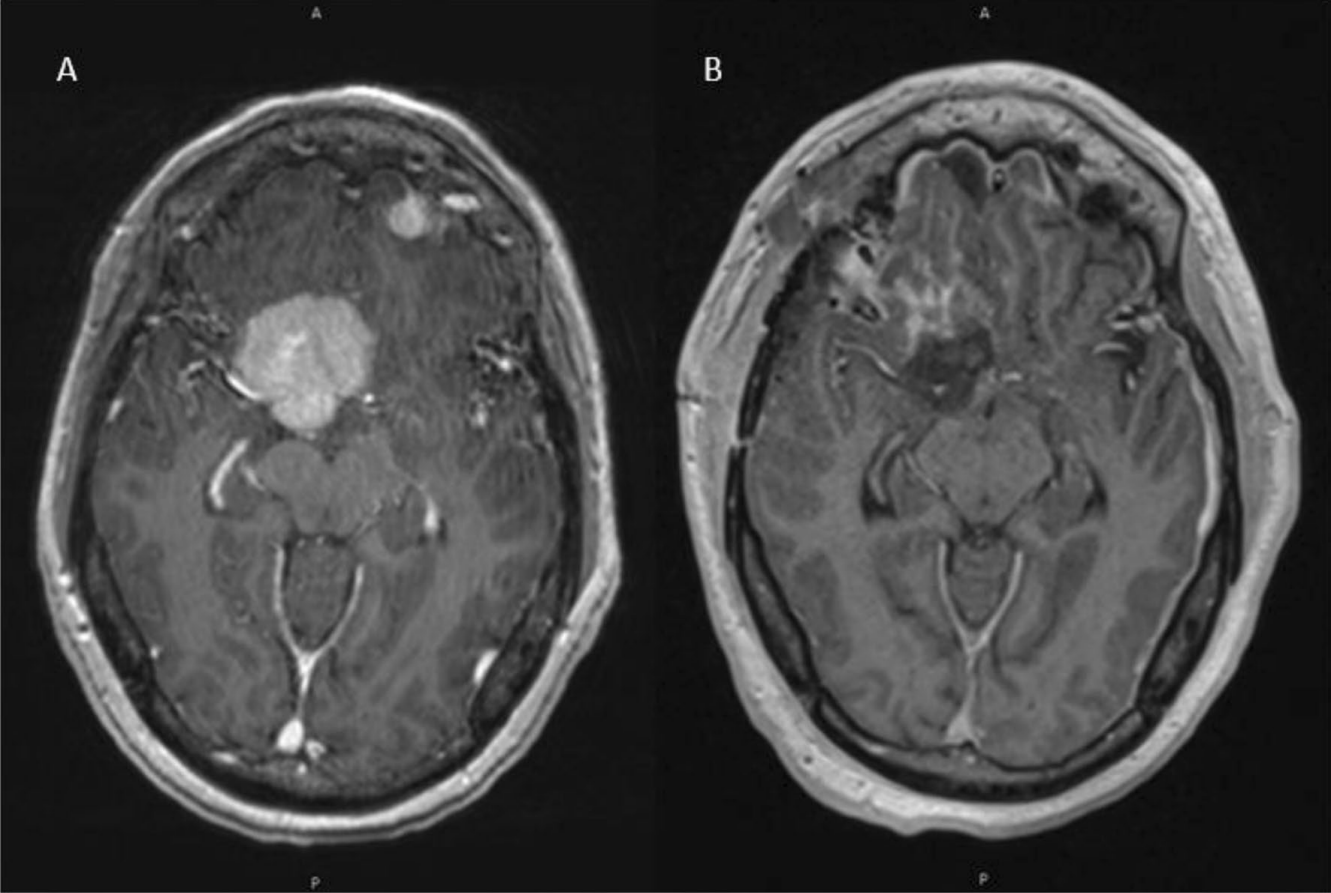
38-Year-old female presented with visual loss in the right eye. Axial MRI with contrast of a patient with a POLR2A somatic drive mutation meningioma located along the planum sphenoidale/tuberculum sellae before (**A**) and after (**B**) resection. Despite its concerning appearance, this tumor is a histologically benign, WHO grade 1 meningioma. However, given the location of the tumor, gross total resection is limited due to surrounding neurovascular structures

Finally, meningiomas originating from the tuberculum sellae region and along the clivus, even extending into the cerebellopontine angle, often harbor *POLR2A* mutations (Fig. 7). While rare, these tumors are associated with benign clinical behavior (Fig. 7) Similar to tumors located along the sphenoid wing, EOR can be limited due to vascular and cranial nerve involvement. Inherent risks in anatomical location, coupled with findings that posterior fossa meningiomas have lower recurrence rates [10], have to be taken into account when considering maximum EOR for these meningiomas.

## Use of molecular data for recurrent meningioma surgery

As the histologic and molecular pathology becomes available following meningioma resection, this information can be helpful in guiding further management by predicting risk of recurrence. In the 2021 WHO Classification of Tumors of the CNS, several molecular features have been incorporated into the pathological grading of meningiomas [12]. Among them, the presence of *TERT* promoter (TERTp) mutations, homozygous deletion of *CDKN2A/B*, BAP1 loss of nuclear expression have all been associated with increased tumor aggressiveness [42, 43]. Epigenetic factors, including DNA and histone methylation profiling, are a recent area of research and have been shown to reliably predict tumor recurrence [44].

TERTp mutant meningiomas are associated not only with increased chance of recurrence after resection but also with molecular transformation, such that tumors are more likely to progress to a higher WHO grade [45]. Patients with meningiomas harboring homozygous *CDKN2A/B* deletions similarly have significantly worse outcomes and more rapid time to recurrence [46]. *CDKN2A/B* allelic status is an independent prognostic factor [42], with the presence of the *CDKN2A/B* homozygous deletion now being an independent criterion for WHO grade 3 classification [12]. Indeed, *CDKN2A* serves as a useful biomarker for identification of meningiomas with a high risk of early recurrence. Complete loss of H3K27me3 is associated with increased risk of recurrence and is an adverse prognostic factor for PFS and OS [47]. Therefore, meningiomas with H3K27me3 loss warrant close follow-up and consideration for adjuvant radiotherapy in cases of STR to reduce the risk of recurrence.

Previous editions of the WHO classification defined both rhabdoid and papillary histologic subtype as an exclusively WHO grade 3 meningioma associated with high rates of recurrence and mortality ^51^. However, recent evidence demonstrates diverse clinical behavior of these histologic subtypes [49]. In the absence of overt high-grade histologic features, some rhabdoid meningiomas have indolent behavior similar to a WHO grade 1 tumor, suggesting underlying genetic factors influencing tumor aggression and clinical course [49]. The BRCA-1 associated protein (*BAP1*) is a deubiquitinating enzyme with a role in tumor suppression, regulating cell proliferation and growth [50]. Somatic *BAP1* mutations are frequently an underlying genomic aberration in rhabdoid meningiomas and are associated with a more clinically aggressive meningioma, resulting in multiple recurrences and shortened OS [51, 52]. Germline *BAP1* mutations have also been identified, increasing the hereditary risk of meningiomas and other cancers including uveal melanoma, cutaneous melanoma, and renal cell carcinoma [53, 54]. Patients with BAP1 rhabdoid meningiomas should be screened for germline BAP1 mutations to determine a predisposed risk of other malignant cancers, both for the patient and family members ^55,56^. If a germline *BAP1* mutation is identified, a thorough cancer history should be performed, and family genetic counseling should be considered to rule out other cancers.

CNV data can help predict tumor behavior with the degree of chromosomal abnormalities strongly associated in recurrence [56–58]. This information is relatively easy to acquire at most centers and enhances its usefulness. Several CNVs are associated with higher-grade meningiomas and include losses of chromosomes 1p, 3p, 4, 6, 10, 14q, 18, and/or 19, with 1p loss being especially suggestive of malignant behavior [56]. WHO grade 1 meningiomas with higher degree of copy number abnormalities have been shown to progress to a higher WHO grade over time [35].

**Fig. 8:**
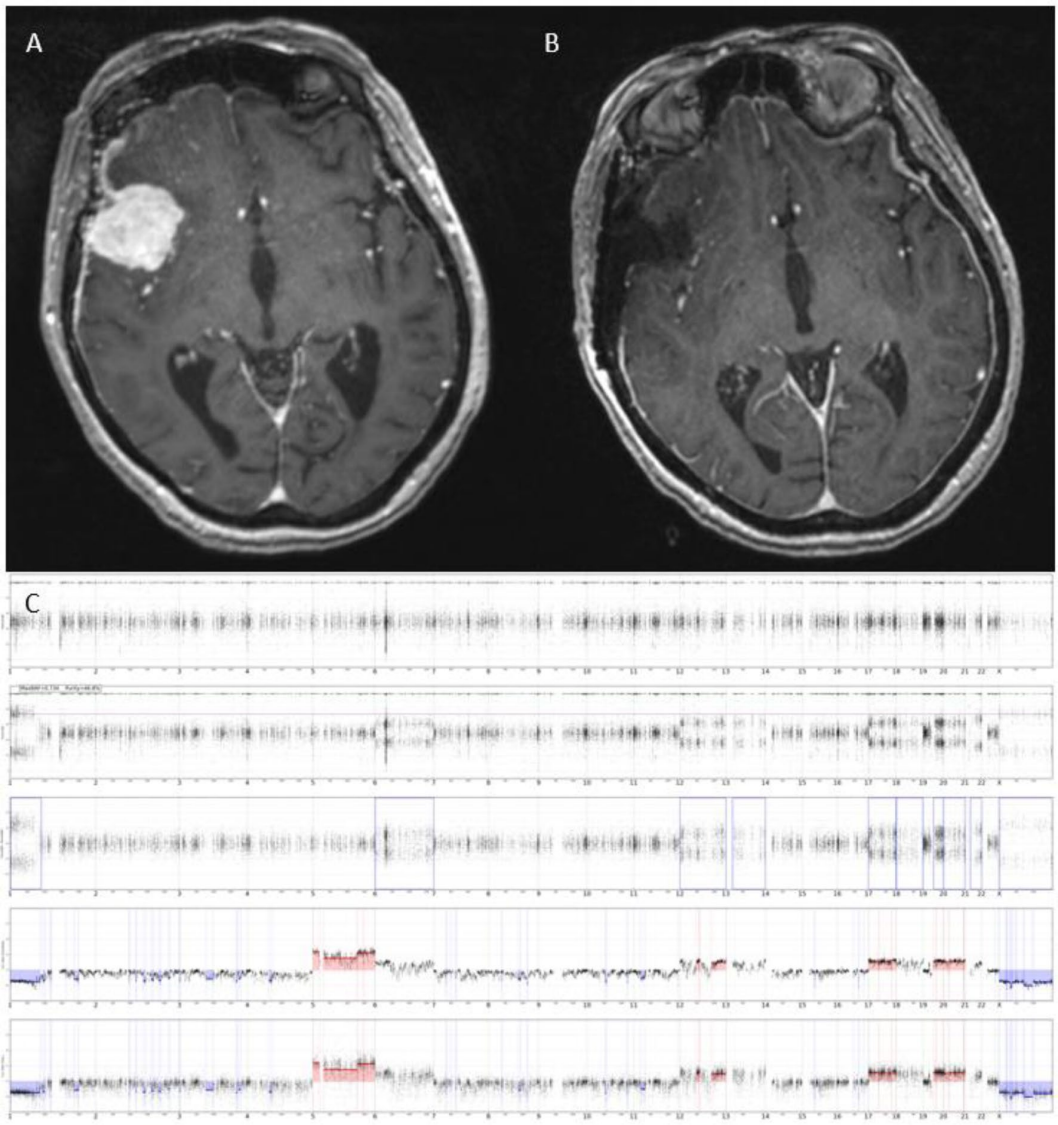
T1 MRI with contrast demonstrating an incidentally discovered right lateral sphenoid wing meningioma before (**A**) and after (**B**) gross total resection. Intraoperative pathology confirmed a WHO grade 1 meningioma. However, further genetic profiling of the tumor demonstrated a more aggressive genomic signature, with a high number of copy number variations (CNV) demonstrated on CNV plot (**C**). The postoperative course of this patient was uncomplicated and they were followed closely with surveillance imaging without receiving radiation therapy

**Fig. 9:**
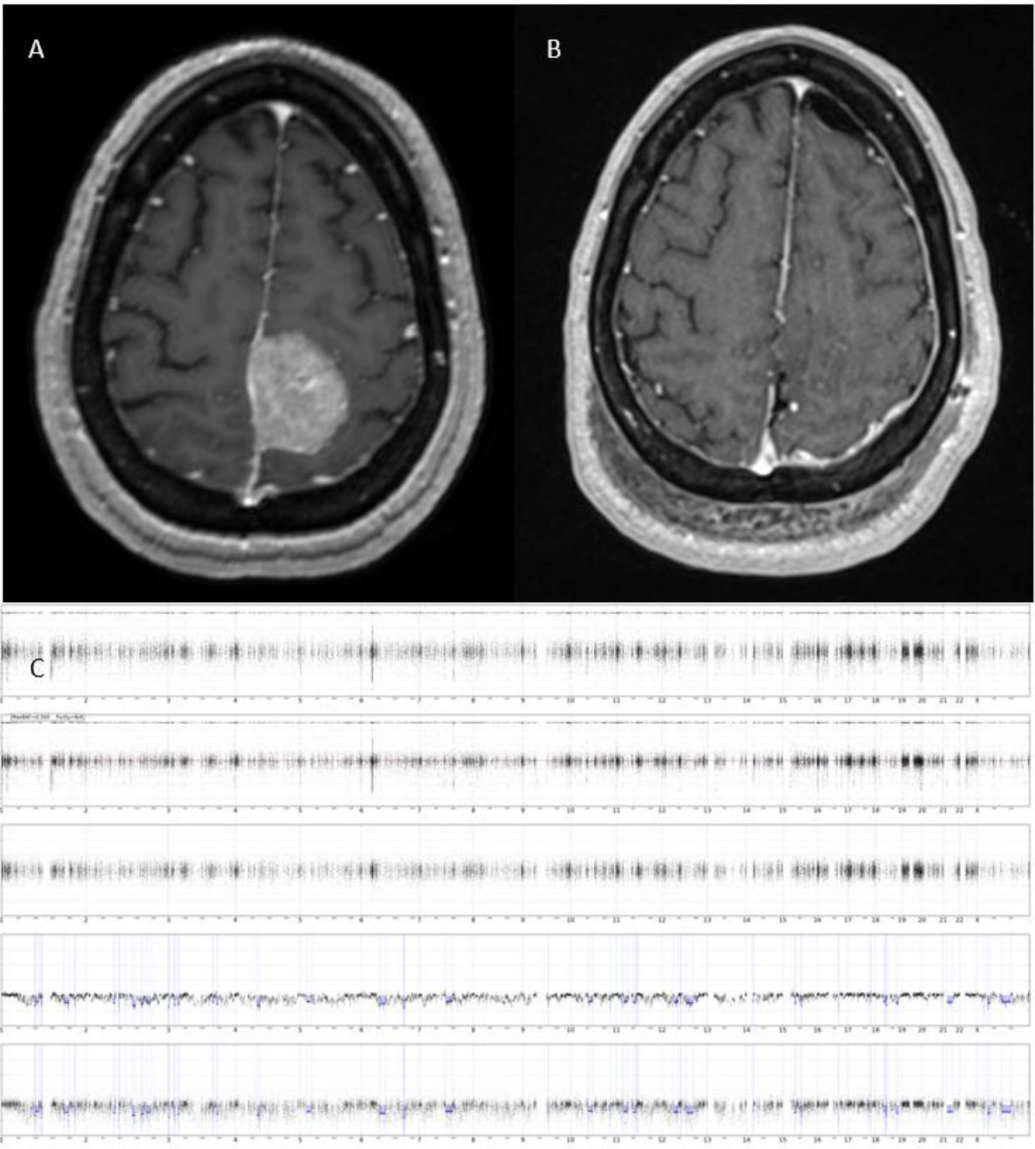
T1 MRI with contrast demonstrating an asymptomatic parafalcine meningioma before (**A**) and after (**B**) gross total resection. Intraoperative pathology confirmed a WHO grade 2 meningioma. However, further genetic profiling of the tumor demonstrated a more benign genomic signature suggestive of a lower-grade meningioma, with no copy number variations (CNV) demonstrated on CNV plot (**C**). The postoperative course of this patient was uncomplicated; following adjuvant radiation therapy, the patient was followed closely with surveillance imaging

Normal or minor copy number alterations not involving the chromosomes identified, monosomy of chromosome 22, and cases with multiple polysomies consistent with angiomatous meningioma are more commonly associated with grade 1 meningiomas and are expected to follow a more “benign” course [56]. While “higher risk” copy number profiles have been reported in 13–29% of grade 1 meningiomas, lesser degrees of percent genome altered are observed in some higher-grade meningiomas (Figs. 8, 9) [56, 58]. These discordances must be considered when making treatment decisions after surgery. Indeed, how the extent of chromosomal abnormalities within the context of a histologic grade should influence the use of post-operative radiation warrants a clinical trial study. However, if a meningioma that has been found to have chromosomal instability recurs, consideration for aggressive surgical re-resection might be justified.

Epigenetic modifications, including methylation patterns, are associated with critical risk stratification and behaviors in meningiomas. A major epigenetic determinant of gene expression and cellular differentiation is the methylation of histones. Modifications to lysine 27 (K27) of histone H3 play a crucial role in tumorigenesis [59], of which trimethylation status (H3K27me3) is of particular importance. H3K27me-negative meningiomas were associated with more aggressive types of meningiomas with increased recurrence in both WHO grade 1 and 2 tumors and worsened OS in grade 3 tumors [43, 47, 60]. DNA methylation profiling has also revealed distinct and clinically relevant methylome-based meningioma subtypes that can predict tumor recurrence and prognosis [61, 62]. Sahm et al. published seminal work describing a DNA methylation-based classification for meningiomas. Performing genome-wide DNA methylation patterns in meningiomas revealed six distinct methylation classes of meningiomas [62]. These methylation patterns correlate with PFS, and more accurately predicted outcome compared to WHO grade.

Building on this work, Nassiri et al. combined DNA methylation profile, WHO grade, and Simpson grade to develop and validate a calculator to predict 5-year recurrence free survival after surgical resection [61]. The calculator was weighted to have methylation profiles as the biggest contribution to predict outcome. The calculator performed as a better predictor of outcome compared to WHO grade alone and was independently associated with recurrence free survival. The development of the calculator allows for individualized decisions to be made for follow-up and post-operative interventions.

DNA methylation profiling is not yet widely adopted, primarily because the therapeutic impact is unclear. Recent work by Choudhury et al. has integrated DNA methylation profiles with genetic, transcriptomic, biochemical, and proteomic factors to identify three distinct DNA methylation groups with distinct clinical outcomes and therapeutic vulnerabilities [44, 56]. The classification scheme identifies meningiomas as Merlin-intact, Immune-enriched, and Hypermitotic. Interestingly, the study found that Immune-enriched meningiomas demonstrated markers of T-cell exhaustion, suggesting that immune checkpoint inhibition would be ineffective against these types of tumors. Additionally, these methylation groups are associated with specific meningioma cell lines that can be used to identify potential chemotherapy targets.

## Conclusions

When surgical resection is indicated, the primary goal of meningioma surgery remains maximally safe resection. For newly diagnosed meningiomas, surgery might be indicated if the tumor is symptomatic, causing significant mass effect, demonstrates interval growth, and/or there is concern for a higher-grade lesion or one associated with more aggressive somatic driver mutations.

While the Simpson Grading Scale has been useful in predicting recurrence or regrowth based on EOR for WHO grade 1 tumors, it remains imperfect and better predictive models are needed to inform patient management decisions. Certain molecular subtypes and methylation statuses indicate aggressive tumor behavior and can predict early progression and recurrence more accurately than Simpson grade and WHO grade.

As we continue to gain mechanistic insights into meningioma genomics and biology, we are refining our management decisions for improved patient outcomes. We can now mostly predict the molecular subtype, and thus aggressiveness, of a meningioma prior to surgery based on characteristics and specific radiographical features, such as tumor location. Postoperatively, in addition to routine histopathologic study, comprehensive tumor genomic profiling is performed in many centers to predict the clinical behavior of meningiomas after surgery. Indeed, an understanding of these molecular and epigenetic features of meningiomas is critical to individualize post-operative decision-making, including the interval for follow-up visits and imaging, for each patient.

Together, these advancements have helped guide surgical strategies and clinical decision making and are allowing for more aggressive and safe surgical resections, and better patient-specific treatment of meningiomas.
